# The Effectiveness of Wearable Devices in Non-Communicable Diseases to Manage Physical Activity and Nutrition: Where We Are?

**DOI:** 10.3390/nu15040913

**Published:** 2023-02-11

**Authors:** Valentina Natalucci, Federica Marmondi, Michele Biraghi, Matteo Bonato

**Affiliations:** 1 Department of Biomolecular Sciences, Division of Exercise and Health Sciences, University of Urbino Carlo Bo, 61029 Urbino, Italy; 2 Department of Infection Diseases, IRCCS San Raffaele Scientific Institute, 20132 Milan, Italy; 3 Department of Biomedical Sciences for Health, Università degli Studi di Milano, 20122 Milan, Italy; 4 IRCCS Istituto Ortopedico Galeazzi, 20161 Milan, Italy

**Keywords:** physical activity, exercise, nutrition, wearable devices, lifestyle assessment, health, non-communicable diseases

## Abstract

Wearable devices are increasingly popular in clinical and non-clinical populations as a tool for exercise prescription, monitoring of daily physical activity and nutrition, and health-related parameters management. In this regard, smart devices not only assist people in pursuing a healthier lifestyle, but also provide a constant stream of physiological and metabolic data for management of non-communicable diseases (NCDs). Although the benefits of lifestyle-based interventions (exercise and nutrition) for NCDs are well known, the potential of wearable devices to promote healthy behaviors in clinical populations is still controversial. In this narrative review, we aimed to discuss the current application of wearable devices in NCDs, highlighting their role in prescribing and monitoring daily physical activity and dietary habits in the population living with chronic diseases. None of the studies considered specifically addressed the efficacy of the use of wearable devices, and limited are those that incorporate monitoring of both physical activity and nutrition for NCDs. However, there is evidence that such devices have helped improve physical activity levels, physical fitness, body composition, and metabolic and psychological parameters. Therefore, the authors believe that the benefits obtained from the use of wearable devices are likely to translate to public health and represent one of the important tools for the development of prevention plans in everyday life and clinical practice for optimal patient management.

## 1. Introduction

The major non-communicable diseases (NCDs), also known as chronic diseases, continue to become exceedingly ingrained in everyday life and represent the main killers in the modern era. Although chronic disease is not contagious, its long duration and progress slowly has a negative impact on the population’s health, accounting for 74% of all deaths globally [1] and representing a high cost to healthcare systems worldwide [2]. The risk factors underlying NCDs include unhealthy behaviors, such as smoking, overeating, alcohol consumption, and sedentary lifestyle, all behaviors that are considered modifiable risk factors. The reversal of these bad health habits can be achieved through leading a healthier lifestyle, with the aim to extend the period of being free from NCDs (primary prevention), to improve managing the disease (secondary prevention), and increasing the quality of life in people with NCDs (tertiary prevention) [3]. To achieve increased adoption of healthy behaviors in the three levels of prevention, integrated action by all clinical and non-clinical stakeholders that looks beyond the traditional model of healthcare and is increasingly in step with the social context is needed.

In the last 10 years, wearable technology has gained in popularity, both in the general population and in people with NCDs, for physical activity and health monitoring, representing nowadays one of the most popular trends in health and fitness [4]. It is well known that physical activity is associated with numerous health benefits, preventing the onset of non-communicable diseases, and resulting in a lower risk of mortality [5]. Recently, Strain et al. [6] showed that higher volumes of device-based physical activity energy expenditure was associated with reduced mortality rates. The public health relevance of such devices is increasing and may impact areas such as physical activity, wellbeing, cardiovascular health, mortality risk, and dietary habits [4]. In fact, commercial wearable devices have the potential to allow for population-level measurement of physical activity and large-scale behavior change. A common example is represented by the self-monitoring of physical activity in terms of step numbers and minutes of daily activity, as well as physiological parameters (e.g., heart rate) or dietary intake (e.g., energy expenditure), depending on the characteristics of the device. Some products also remind one to stop sedentary activities or introduce meals through notification messages [7]. All this data can be used by users to plan, organize, and monitor their daily physical activity or dietary habits, but can also provide immediate feedback that can eliminate the barriers between the patient and healthcare professional. Moreover, wearable technology can also aid researchers in planning study designs with the aim to use physical activity monitor devices, especially in people with NCDs [8].

In this regard, the actual wide use of wearable devices connected to the internet, social media, and databases, increases the use of real-world data in the medical and healthcare field to monitor patient health status [9]. In fact, new advances in technology for objective assessment of physical activity using wearable devices in research enable testing the efficacy of interventions and give the opportunity to prescribe and monitor patients’ home-based physical activity and its effect on health. As technology advances, these devices took the top spot in the American College of Sports Medicine (ACSM) list in 2017, 2019, 2020, and 2022, with nutrition devices also growing in popularity over the past 3 years [10,11,12].

Although the use of wearable devices has consistently entered society, currently there is a lack of sufficient evidence and data on how wearable devices can be implemented in the NCDs population in both the clinical and non-clinical setting. Therefore, the aim of this narrative review is to discuss the current application of wearable devices in NCDs, highlighting their role in prescribing and monitoring daily physical activity and dietary habits in the population living with chronic diseases. In the following sections, we will first provide an overview of the relationships between NCDs and major, modifiable, behavioral risk factors, such as exercise and nutrition. We will examine the utility of wearable devices for prescribing and monitoring modifiable lifestyle behaviors (exercise and nutrition) and provide an overview of randomized-controlled studies that have included the use of wearable devices for prescribing and monitoring exercise and nutrition habits in the population living with NCDs. We will end with a discussion of the effectiveness and potential role of emerging technologies for prescribing and monitoring daily physical activity and dietary habits, in addition to discussion of the barriers to the integration of the technology into the population and into research and clinical settings.

## 2. Relationships between Non-Communicable Diseases and Modifiable Behavioral Risk Factors

### 2.1. Sedentary Risks or Unhealthy Physical Activity Levels

In clinical and non-clinical populations, insufficient physical activity levels in all stages of life are a well-documented public health issue [13]. Between 2001 and 2016, the levels of insufficient physical activity decreased marginally globally in high-income countries, which reported a 5% increase in the prevalence of physical inactivity [14]. Moreover, during the COVID-19 pandemic, due to contagion reduction policies based on home confinement and social distancing, a reduction of 33% from 108 to 72 min per week and an increment of 28% of time spent sitting from 5 to 8 h per day was observed in different chronic diseases [15,16,17,18]. High levels of sedentary behavior have a negative impact on the human body at the level of the muscular, cardiovascular, metabolic, endocrine, and nervous systems [19], resulting in an increased risk of developing a variety of chronic diseases, including cardiovascular diseases, type 2 diabetes, cancers, and mental illness [20]. To date, people with low levels of physical activity use significantly more healthcare services than active people [21]. The novel aspect to counteract the extraordinary burden of insufficient physical activity and improve understanding of its negative effects lies in the application and monitoring of physical activity components in a new human evolutionary context different from that of our ancestors (hunter-gatherers), who were forced to be physically active to survive.

Physical activity is defined as “any bodily movement produced by the contraction of skeletal muscles that results in energy expenditure” [22]. In this regard, growing evidence suggests a clear dose–response relationship between the volume of physical activity and the difference in mortality rates for chronic disease [23]. The updated current adults physical activity guidelines of the World Health Organization (WHO) recommend undertaking 150–300 min of moderate intensity or 75–150 min of vigorous intensity aerobic physical activity, or some equivalent combination of moderate-intensity and vigorous-intensity aerobic physical activity per week [24]. The same guidelines suggest that adults should also perform moderate- or higher-intensity muscle-strengthening activities involving all major muscle groups 2 or more days a week. In addition, an increase in attention is also directed to daily steps as an important part of preventing chronic disease [25,26,27,28]. Even if the guidelines refer to the general healthy population for primary prevention, different thresholds have been recommended for secondary and tertiary prevention for different chronic conditions considering the several components (i.e., the FITT principle [frequency, intensity, time, and type]) of physical activity. There is currently a lack of knowledge regarding the minimum amount of exercise needed to prevent and mitigate different chronic diseases, but promising evidence exists on how individual components of FITT affect the development and progression of chronic diseases. Today, we can talk about ‘exercise medicine’, in which the response to exercise is specific and predictable for optimal adjuvant treatment of over 30 chronic diseases [29]. The expanded understanding of exercise physiology and molecular biology has provided strong evidence on how different components and combinations of exercise (i.e., frequency, intensity, time, and type of exercise) may play a critical role in the persistent, low-grade inflammation that characterizes chronic diseases [30,31,32,33,34]. Evidence from epidemiological studies and clinical research demonstrates that the exercise component also exerts its effects on mental diseases. As with other chronic diseases with an impact on physical function, mental diseases include symptoms that, while they are different for everyone, can benefit from exercise [35,36,37].

Although the link between exercise and signaling pathways at the cellular and molecular levels can induce beneficial effects on physical and mental health, more than 80% of people do not meet physical activity guidelines [38].

### 2.2. Dietary Risks or Unhealthy Foods Consumption

When macronutrients are consumed in appropriate proportions to support energetic and physiologic needs without excess intake, while also providing sufficient micronutrients and hydration to meet the physiologic needs of the body, it is called a ‘healthy diet’ [39]. However, over the past 40 years, population diets have shifted toward a greater consumption of processed and ultra-processed foods that are low in nutrients and high in energy [40], with major public health consequences [41].

Unhealthy diet patterns, including the intake of foods high in saturated fat, refined carbohydrates, and excess sodium, coupled with lower physical activity levels, have resulted in higher prevalence rates of obesity, cardiovascular disease, type 2 diabetes, site-specific cancers, and respiratory diseases [42,43,44,45,46,47]. According to data from the NCD Risk Factor Collaboration and the Global Burden of Disease Study, NCDs, including those listed above, are responsible for almost 70% of all deaths worldwide, while changes in eating habits play a crucial role in preventing up to 80% of major NCDs [48].

In the modern era, unhealthy nutritional behaviors continue to be recorded in people with and without NCDs. These behaviors are not only limited to extreme social conditions, such as the latest COVID-19 pandemic [49]. Indeed, there is compelling evidence that diet can be influenced by many social and economic factors that evolve over time and interact in a complex manner (e.g., income, food prices, individual preferences and beliefs, cultural traditional, geographical, and environmental aspects). While there is no clear model that can pinpoint the people who adopt an unhealthy diet, on the other hand, the effects that the high consumption of trans-fatty acids has on the deterioration of health conditions are well document. The first consequence is low-grade systemic inflammation, which is positively correlated with type 2 diabetes, cardiovascular disease, cancer, and premature death [50,51]. In addition, particular attention is also paid to the consequences of intra-abdominal fat accumulation as an important risk factor for the promotion of insulin resistance, which can lead to glucose intolerance, elevated triglycerides, and low high-density lipoprotein, as well as hypertension [52,53,54].

With the aim to counter global unhealthy diets and to prevent a range of NCDs, WHO has developed evidence-based guidelines [55]. As with the exercise guidelines, the exact composition of a healthy diet also depends on individual characteristics (e.g., age, gender, lifestyle, and level of physical activity), cultural context, locally available foods, and dietary customs. Following a healthy diet could be easier in those countries where certain nutritional models, such as the Mediterranean diet (i.e., Euro-Mediterranean countries), are rooted. Paradoxically, that is not the case, and there is compelling evidence that most people from industrialized countries do not meet the current dietary recommendations [56].

## 3. The Utility of Wearable Devices on Lifestyle Behaviors

The use of smart technology has a dual role in the clinical and non-clinical context (healthcare and social). Particularly, it represents an important prevention tool, characterized by the possibility of the individual to measure and monitor health parameters because of his lifestyle-related behaviors. Furthermore, it also represents an important continuous monitoring tool, through which clinicians and non-clinician professionals can personalize the exercise prescription and nutritional intake of the individual living with or without NCDs.

### 3.1. Wearable Devices for Prescription and Monitoring Physical Activity

To counteract sedentary behavior, defined as an insufficient physical activity level, according to WHO recommendations, is physical activity. Humans’ evolutionary history suggests that today, people must be active, but paradoxically find themselves in an increasingly industrialized context, forced to be sedentary [57]. In fact, the effects of the competing demands of activity and inactivity on the body represent an evolutionary paradox that could be countered by the evolutionary thrust of technology.

Advances in 21st century technology have introduced the use of commercial off-the-shelf activity trackers that allow users to self-monitor their daily physical activity [58]. These devices allow objectively monitoring physical activity using several consumer-based activity trackers equipped with different sensors, including accelerometers, heart rate (HR), Global Positioning System (GPS) technology, gyroscope, barometer and altimeters, and algorithms built into smartphones or wearable technology (e.g., bracelets, bands, rings, etc.), which determine a device output, such as step-count, distance traveled, energy expenditure, activity intensity, and HR [59]. This need arises from the consequences that self-reported questionnaires and diaries suffer from participants’ biases as self-report measures, such as social desirability or imprecise recall [60]. According to Chan et al. [61], wearable devices represent a promising intervention tool for population-wide physical activity promotion, providing real remote monitoring of users’ physical fitness. In particular, activity trackers offer a significant source of personalized physical activity data from users that would provide insights into healthcare analytics and user feedback on health status, assisting clinicians and experts in the field (e.g., sport scientists) in providing a more holistic care [62].

### 3.2. Wearable Devices for Prescription and Monitoring Dietary Intake

As with exercise, wearable technology, websites, and smartphone applications could estimate the nutritional intake of individuals. In the modern era, the self-reported method using smartphones has discouraged the use of conventional paper-based self-assessment methods both for more practical reasons and for reasons of data accuracy [63,64,65]. The network capacity of a smartphone also allows this modern approach to have more features, such as a response from clinicians and real-time self-monitoring [66]. The new systems allow participants to record entire days of meals and to monitor their intake of kilocalories and macronutrients. In addition, depending on the applications, there are also other features that allow the consumer to set goals, plans meals, or even track micronutrients.

Over time, research has also demonstrated that the timing and frequency of self-monitoring dietary intake are significantly related to weight outcomes [67]. These findings related to the importance of the frequency of self-monitoring within each day, as well as the consistency throughout a month, amplify the importance of increasingly accessible and practical tools that enable the development of very specific self-monitoring dietary goals.

### 3.3. Wearable Devices in Non-Communicable Diseases: Where We Are

In the last decade, researchers have begun to take advantage of this wearable technology by incorporating these devices in their research studies.

**Figure 1:**
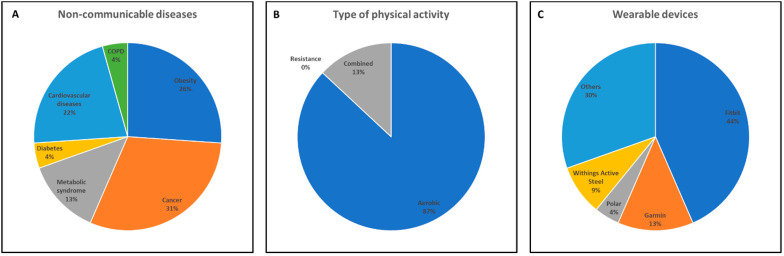
Pie charts of non-communicable diseases (**A**), type of physical activity (**B**), and wearable devices (**C**) used in the RCTs from 2016 to 2022.

In this regard, here we have reviewed all the longitudinal RCTs from 2016 to 2022 that used wearable devices, focusing on the outcomes that could be relevant for improving physical activity and health-related outcomes in people with NCDs (Table 1). To date, 23 RCTs have been published [68,69,70,71,72,73,74,75,76,77,78,79,80,81,82,83,84,85,86,87,88,89,90]. The NCDs studied were cancer (31%), obesity (26%), cardiovascular diseases (22%), metabolic syndrome (13%), diabetes, and COPD (4%) (Figure 1A). Study protocols had a mean duration of 22 weeks, ranging from 2 to 96 weeks. Participants were a mean of 59 years old, ranging from 31 to 72 years old. The physical activity protocols performed during the RCTs were mainly aerobic (87%), followed by the combination of aerobic and resistance training (13%). None of the RCTs analyzed used a resistance training protocol alone (Figure 1B). The wearable devices used during the studies were Fitbit (44%), Garmin (13%), Polar (9%), and others (30%) (Figure 1C). Drop-out rates during the studies were a mean of 11%, ranging from 0% to 60%. Analyzing the RCTs, all the physical activity protocols showed an increase of physical activity levels with an increase of self-reported physical activity, daily steps, moderate to vigorous physical activity, and motivation to perform physical exercise, with a general reduction of sedentary time. This increase in physical activity levels improved cardiorespiratory fitness, with a reduction of the parameters associated with body composition (body weight, body mass index, fat mass, and waist circumference) and metabolic parameters (glycated hemoglobin and systolic and diastolic blood pressure), and an improvement in psychological parameters (anxiety and depression scores). Regarding nutrition, only eight studies considered this aspect. Particularly, 3 studies monitored diet during the experimental protocol using a 3-day [77] or 7-day food diary [71,79]. Three studies provided nutritional counselling for self-monitoring food intake, planning specific educational sessions with weekly and monthly phone recall [69,82,87]. One study assessed with a specific questionnaire the adherence to the Mediterranean diet [88]. Finally, only one study prescribed a calorie-restricted diet. To date, this was linked with an improvement of body composition and metabolic parameters in obese postmenopausal women [86].

## 4. Discussion

This review analyzed 23 RCTs in a narrative manner to highlight the role of wearable devices in prescribing and monitoring daily physical activity and dietary habits in people with NCDs. In particular, it emerged that the prevalent use of wearable devices is aimed at the prescription and monitoring of the physical activity protocol to increase physical activity levels. Wearable devices have been used to prescribe and monitor different types of exercise. As reported in Figure 1B, aerobic exercise was prescribed the most (87%) [69,70,71,72,73,74,75,76,78,79,80,81,83,84,85,87,88,89,90], followed by combined exercise (13%) (i.e., aerobic and resistance) [68,77,82,86]. However, it is well documented in the literature that resistance exercise alone can bring about significant health changes in numerous chronic conditions [91]. This lack, which we find in the studies analyzed, could depend on the current low propensity of wearable devices to monitor functional parameters, such as the muscle strength of the lower and upper limbs. In fact, although by apps it is possible to prescribe resistance exercises to the subject, monitoring is poor due to the lack of accurate wearable devices. To date, the main specific metrics that can be derived from exercise monitoring wearables are shifted towards physiological parameters, such as the number of steps, distance traveled, and cardiometabolic parameters (e.g., HR, energy expenditure, maximum oxygen consumption, oxygen saturation, and blood pressure).

In pursuing physical activity with health implications, tracking daily step count is an invaluable component. The Fitbit was the most used wearable technology in the studies analyzed (Figure 1C). However, as reported in a 2020 systemic review [8], wearable devices are accurate for measuring step count in the laboratory, but exhibit a wider range of inaccuracy in free-living environments. In addition, another major problem is the estimation of the number of steps: some devices overestimate, while others underestimate, and such variability in terms of reliability exists intra-device (i.e., the step count may differ not only within the same company, but also within the same device).

These issues were also found in standard measurements of maximum oxygen consumption (*V*O_2max_). Though *V*O_2_ estimation has become more common in wearable devices, the validity and reliability among wearable devices measuring this parameter is still controversial, and none of the popular smartwatches has been well validated [4,92]. If we consider that *V*O_2max_ has proven to be a powerful indicator of health and has recently been proposed as a clinical vital sign by the American Heart Association [93], it is disheartening to note that among the studies analyzed, this parameter was only considered in one study [90]. In fact, the major outcomes of the studies analyzed were: (i) number of daily or weekly steps [70,72,74,75,77,78,83,84,85,87,89]; (ii) physical activity levels in terms of intensity increments (e.g., from moderate to vigorous), total minutes of daily activity, reduction in sedentary time, and daily walking time [68,69,71,73,76,77,80,81,87,88]. In two studies, the outcomes were also measured subjectively through questionnaires for daily physical activity levels: the Freiburger Physical Activity Questionnaire and work ability index [71] and the Walking Impairment Questionnaire [86].

Less commonly, clinical outcomes, such as blood pressure [77], metabolic syndrome [79], and physical function [82,83], or psychological parameters, such as anxiety and depression [88] and exercise motivation [81], were explored.

The selection of suitable activity trackers in populations living with NCDs should be able to offer patients and healthcare professionals the reading of the main outcomes of interest that should be monitored for health purposes, without excluding anyone. Therefore, collaboration with activity tracker companies to overcome existing discrepancies remains crucial and should focus on the accuracy and validity of the device. In fact, the accuracy in the monitoring of the different parameters needs to be more precise to collect the physiological and activity outcomes. While wearable activity trackers offer considerable promise for helping people’s lifestyle behaviors, there are numerous barriers researchers and users find when using them. First, prolonged use of activity trackers may cause study participants to experience behavioral problems, such as health status monitoring-induced anxiety. Secondly, breakage or loss of the device and technical difficulties with the accompanying device/software were noted as other barriers [94]. Consequently, these barriers may negatively impact study participants’ adherence to wearing trackers [95]. Another important barrier is also represented by the reading of the results themselves. In fact, understanding the results could be an incentive for wearable device users, while, as highlighted in a survey, understanding the data is often unknown. Device users find themselves asking for the help of a physician or health coach to guide them in understanding the data of their wearable technology to make lifestyle changes. Moreover, they are often willing to pay for this service, as well [4]. In the field of exercise, an expert in sport sciences with a master’s degree in Preventive and Adapted Physical Activity is essential for the correct prescription and correct monitoring of physical activity in population living with NCDs. His role lies in being able to accompany the subject with chronic disease step by step in achieving his health goals by managing the continuous comparison and readjustment of the exercise proposals. Furthermore, it can represent valid support both for healthcare professionals (medical specialists) and for the patient in reading the objective data derived from wearable devices.

Regarding nutrition, only eight studies [69,71,77,79,82,86,87,88] considered and analyzed nutritional aspects in their trials. Unlike exercise prescription and monitoring, nutritional aspects were predominantly delivered through face-to-face or remote (e.g., by telephone) educational counselling and monitored through a food diary analyzed and reviewed by dietitians. The lack of precision control for eating habits certainly represents a bias in reading the results. In fact, poor monitoring of nutritional aspects can influence the observation of possible improvements in physical fitness, body composition, and metabolic parameters. In this regard, articles that have considered nutritional aspects, in addition to physical activity, have stated that this combination can lead to greater health outcomes than those of exercise alone or nutrition alone. Therefore, accurate and objective monitoring of both lifestyle aspects is desirable for the development of appropriate health promotion interventions.

To date, evidence indicates that wearable devices have not yet reached such a level of development to effectively help researchers or clinicians in monitoring nutrition and, consequently, caloric intake. For both exercise and nutritional aspects, collaboration and regular feedback from exercise specialists and dietitians with other health professionals and device users is still critical to accurately monitoring lifestyle behaviors. For this reason, an integration between health and non-health professionals should be encouraged to limit this shortage and promote a healthy lifestyle in people living with chronic diseases.

## 5. Current Lifestyle Models and Future Directions

Despite the benefits of a healthy lifestyle based on correct physical activity levels and healthy foods being well documented, an unhealthy lifestyle is rapidly becoming a major global concern, with health, economic, environmental, and social consequences [96]. Nowadays, any lifestyle intervention should consider physical activity and nutrition as two parallel lines of intervention. The first intervention on physical activity has a twofold objective: the first one aims to reduce sedentary behavior, with daily strategies for carrying out physical activity (e.g., using the stairs instead of the lift, parking away from the workplace, etc.); and the second aims to introduce a structured exercise program into daily life with specific goals [28]. Similarly, the intervention on nutrition also has a twofold objective: the first aims to introduce healthy foods into everyday life; and the second aims to introduce a nutritional program with specific objectives in the presence of NCDs.

The emerging literature contains many examples of interventions, in which physical activity and nutrition are part of the same model, some of which are applied to populations living with NCDs, and the results demonstrate that this can be a model to scale up [97,98,99]. The success and feasibility of introducing this new model of care, which complements standard care, requires the implementation of specific action plans that may differ from country to country. This may depend on who is authorized to prescribe the exercise or diet, but also on the health system of the country where the model is to be implemented. In this regard, to be effective, this model needs to overcome the barriers of each country and become integrated into a delivery system; this is often guaranteed by the involvement of different health professionals (general practitioners, specialist doctors, dietitians, exercise specialists, and psychologists), who create the so-called multidisciplinary team, in which technology also plays an important role. However, in many countries, benefits stop outside research programs.

To bypass this problem, healthcare institutions and policy makers should make it easier to use wearable devices, which are often very expensive, and make the reality of a multidisciplinary team at the service of the patient more robust. The results of this narrative review may be useful for the development of new remote healthcare services via mobile devices that researchers or clinicians could consider for population health.

## 6. Strengths and Limitations

This is an emerging field of investigation, and our RCT analysis work provides the best evidence, to our knowledge, to shed light on the benefits of wearable devices in NCDs to date. Compared to other studies, our approach takes a holistic view of wearable device usage. Indeed, we reviewed the two most important lifestyle aspects (exercise and nutrition) to enable: (i) a better understanding of the use of wearable devices, in NCDs, in lifestyle change; and (ii) the development of new questions for future research. In this regard, a strength of this work is that it can help advance the field by clarifying the use of different devices in prescribing and monitoring healthy lifestyles and by offering, albeit preliminarily, sufficient data to understand the applicability to the different NCDs, on which wearable devices for monitoring and prescribing healthy behaviors have already been applied. The overview provided a solid foundation, upon which to direct future studies to achieve health improvements in clinical populations and age groups. The magnitude of the strength of this literature review is of clinical significance, and based on early results, the gains appear to be promising. We also have some limitations of our work. First, this is a narrative and not a systematic review; therefore, it did not allow drawing firm conclusions on the efficacy of wearable devices to prescribe and monitor physical activity protocols and nutritional aspects to reach beneficial health effects. Second, studies utilizing smartphone apps are not incorporated in this review. Although smartphone apps are gaining much attention in research, many are still not validated for usage in scientific research [100].

## 7. Conclusions

With the growing interest in the use of wearable devices and the global need to reduce the health burden of people living with chronic diseases, this study highlighted that more efforts are needed for wearable technologies to integrate them reliably into the field of prevention.

This study has shown that wearable devices have the promise to be an effective method for physical activity monitoring for the prevention, treatment, and improvement of the health and quality of life in people with NCDs. However, the devices are predominantly used to monitor and intervene in physical activity, rather than other daily activities (e.g., diet and sleep) or physiological parameters. Taken together, findings support wearable devices as a valuable tool to reduce the workload of medical personnel and hospitals, allowing, where possible, autonomous management of daily habits in terms of physical activity and nutrition. In the near future, there is a need for innovative measurement tools that are accurate, reliable, and effective for both exercise-related and nutritional aspects, as well as better training for their use. To date, these devices cannot do that without a continuous dialogue between the patient and healthcare professionals.

## Figures and Tables

**Table 1:** Summary of the exercise- and nutrition-based intervention and wearable devices for health-related outcomes of the included studies.

Author, Year [Reference]	Study Design (Duration)	Sample Size (Non-Communicable Disease)	Age (Years)	Exercise-Based Interventions	Nutrition Counselling/ Monitoring	Exercise Prescription/ Monitoring (Wearable Device)	Results
Alley et al., 2022 [68]	RCT (12 weeks)	243 (obese people)	69 ± 4	Tailored advice only (*n* = 96): web-based program with 6 modules of tailored advice delivered biweekly for reaching 30 min of moderate-intensity physical activity on at least 5 days each week, including 2 to 3 sessions of resistance and flexibility activity. Tailoring + Fitbit (*n* = 78): same protocol but with use of the wearable devices. Control group (*n* = 69): usual care.	Not considered	Fitbit	(i) Drop out: 60%. (ii) Tailored advice only: ↔ moderate to vigorous physical activity. (iii) Tailoring + Fitbit: ↑ moderate to vigorous physical activity. (iv)All groups: ↑ self-reported physical activity.
Ferrante et al., 2022 [69]	RCT (12 months)	44 (African American/Black women breast cancer survivors)	21–75 *	5% wight loss goal, 1200–1500 kcal daily, and 10 to 30 min per day of moderate physical activity and 10,000 steps per day (*n* = 44). Fitbit only (*n* = 17). Fitbit + SparkPeople (*n* = 17). Fitbit + SparkPeople Premium (*n* = 10).	Participants with SparkPeople device were educated for self-monitoring nutrition and weigh tracking.	(a) Fitbit (b) SparkPeople	(i) Drop out: 0%. (ii) Devices helped to improve activity levels.
Agarwal et al., 2021 [70]	RCT (12 weeks)	180 (obese people)	56 ± 13	12-week game with points and levels designed using behavioral economic principles to reach step goals (*n* = 180). Gamification with social support group (*n* = 60). Gamification + financial incentives group (*n* = 60). Control group (*n* = 60).	Not considered.	(a) Fitbit (b) Inspire (c) Way to Health platform	(i) Drop out: 1%. (ii)Gamification with social support group: ↔ daily steps. (iii) Gamification + financial incentives group: ↑ daily steps. (iv)Control group: ↔ daily steps.
Haufe et al., 2021 [71]	RCT (6 months)	314 (people with metabolic syndrome)	47 ± 8	Exercise group (*n* = 160): personal counselling with recommendations aiming to perform 150 min of moderate–intense physical activity per week. Control group (*n* = 154): usual care.	Exercise group completed a 7-day food diary which was analyzed and reviewed by dietitians for macronutrient and micronutrient content using professional nutrition analysis software.	Garmin Forerunner 35	(i) Drop out: 13%. (ii) Exercise group: ↑ Questionnaire-estimated exercise activities; ↑ maximum power output. (iii) Control group: not applicable.
Patel et al., 2021 [72]	RCT (12 months)	361 (people with type-2 diabetes)	53 ± 10	Conducted goal setting and entered a 1-year game designed using insights from behavioral economics with points and levels to reach step goals and weight loss targets (*n* = 361). Gamification with support (*n* = 92). Gamification with collaboration (*n* = 95). Gamification with competition (*n* = 87). Control group (*n* = 87).	Not considered.	Withings Activite Steel	(i) Drop out: 7%. (ii) Gamification with support, Gamification with collaboration, Gamification with competition and Control group: ↑ mean daily steps; ↓ weight; ↓ glycated hemoglobin.
Hardcastle et al., 2021 [73]	RCT (12 weeks)	68 (cancer survivors)	64 ± 8	Intervention group (*n* = 34): reducing bouts of sedentary behavior and responding to the automatic Fitbit prompts to take steps, in addition to encouraging planned bouts of moderate to vigorous physical activity. Control group (*n* = 34): only received printed materials containing the physical activity guidelines.	Not considered.	Fitbit Alta	(i) Drop out: 6%. (ii) Intervention group: ↑ moderate-to-vigorous physical activity. (iii) Control group: ↓ moderate-to-vigorous physical activity.
Pinto et al., 2021 [74]	RCT (12 weeks)	20 (older (65+ years) cancer survivors)	72 ± 4	Adjust step goals every week (*n* = 20). Audiobook group (*n* = 12). Comparison group (*n* = 8).	Not considered.	(a) Fitbit Charge 2 (b) Hoopla	(i) Drop out: 5%. (ii) Audiobook group: ↑ steps per day. (iii) Comparison group: ↔ steps per day.
Chen et al., 2021 [75]	RCT (24 weeks)	602 (obese people)	39 ± 10	Strive for their daily step goal in which participants compete against each other or work together depending on group (*n* = 602). Class 1 (*n* = 328): more extroverted and more motivated; had previously used a wearable device. - Control group (*n* = 71). - Gamification with support (*n* = 81). - Gamification with collaboration (*n* = 86). - Gamification with competition (*n* = 90). Class 2 (*n* = 121): less active and less social; never used a wearable device. - Control group (*n* = 33). - Gamification with support (*n* = 30). - Gamification with collaboration (*n* = 29). - Gamification with competition (*n* = 29). Class 3 (*n* = 153): less motivated and at risk. - Control group (*n* = 47). - Gamification with support (*n* = 40). - Gamification with collaboration (*n* = 35). - Gamification with competition (*n* = 31).	Not considered.	Withings Activite Steel	(i) Drop out: 2%. (ii) Class 1: ↑ mean daily step counts in the gamification + competition arm. (iii) Class 2: ↑ mean daily steps relative to control during the intervention period. (iv) Class 3: ↔ mean daily steps relative to control for any of the gamification arms.
Peacock et al., 2020 [76]	RCT (12 months)	204 (people with cardiovascular disease and/or type II diabetes)	64 ± 6	Intervention group (*n* = 134): Personal multidimensional aerobic physical activity feedback using a customized digital system and app for 3 months, plus 5 health trainer-led sessions. Control group (*n* = 70): usual care.	Not considered.	(a) BodyMedia Core (b) SenseWear^®^ Pro 8.0	(i) Drop out: 10%. (ii) Intervention group and Control group: ↔ mean physical activity levels.
Roberts et al., 2019 [77]	RCT (8 weeks)	40 (adults with coronary artery disease events)	70 ± 7	Exercise group (*n* = 20): aerobic and resistance exercises, twice weekly. Counselling on reducing sedentary behavior and increasing non-exercise physical activity. Exercise + non-exercise physical activity (*n* = 20): also tracking non-exercise physical activity with Fitbit.	All participants performed a 3-day diet recall.	(a) Polar Ft2 (b) Fitbit Zip	(i) Drop out: 10%. (ii) Exercise group: ↑ daily steps; ↓ sedentary time; ↓ systolic and diastolic blood pressure. (iii) Exercise + non exercise physical activity: ↔ in all outcomes.
Singh et al., 2020 [78]	RCT (12 weeks)	52 (women with stage II-IV breast cancer)	51 ± 9	Physical activity counselling (*n* = 26): physical activity levels, moderate to vigorous physical activity, were assessed using physical activity counselling and surveys; 150 min physical activity per week. Physical activity counselling + Fitbit (*n* = 26): also received an activity tracker.	Not considered.	(a) Fitbit Charge HR (b) Actigraph^®^ GT3X+	(i) Drop out: 4%. (ii) Physical activity counselling: ↔ steps/day. (iii) Physical activity counselling + Fitbit: ↑ steps/day during moderate to vigorous physical activity.
Haufe et al., 2019 [79]	RCT (6 months)	314 (people with metabolic syndrome)	48 ± 8	Exercise group (*n* = 160): personal counselling with recommendations aiming to do 150 min of moderately intense physical activity per week. Control group (*n* = 154): usual care.	All participants completed a 7-day food diary, which was analyzed and reviewed by dietitians for macronutrient and micronutrient content. All participants in the exercise group received nutritional counselling, which provided background information on healthy food choices.	(a) Garmin Forerunner 35	(i) Drop out: 13%. (ii)Exercise group: ↓ metabolic syndrome severity. (iii) Control group: ↔ metabolic syndrome severity.
Lynch et al., 2019 [80]	RCT (12 weeks)	83 (women with stage I–III breast cancer)	62 ± 6	Intervention group (*n* = 43): face-to-face session. Acoustic and visual alerts for inactivity were set. Telephone-delivered behavioral counselling. Control group (*n* = 40): usual care.	Not considered.	(a) Garmin Vivofit 2 (b) Actigraph (c) ActivPAL	(i) Drop out: 4%. (ii) Intervention group: ↑ levels of moderate to vigorous physical activity. (iii) Control group: ↔ levels of moderate to vigorous physical activity.
Van Blarigan et al., 2019 [81]	RCT (2 weeks)	42 (people with colorectal cancer)	54 ± 11	Intervention group (*n* = 21): 150 min/week of moderate activities or 75 min/week of vigorous activities; 2–3 times per week. Control group (*n* = 21): received print educational materials about physical activity after cancer.	Not considered.	(a) Fitbit Flex™ (b) ActiGraph GT3X+	(i) Drop out: 7%. (ii) Intervention group: ↑ activity levels; ↑ motivation to exercise. (iii) Control group: ↑ activity levels.
Lee et al., 2019 [82]	RCT (12 weeks)	96 (prostate cancer patients)	69 ± 7	Intervention smartphone group (*n* = 50): home-based aerobic and resistance exercises, provided with Smart After-Care app and a wearable InbodyBand digital pedometer. Pedometer control group (*n* = 50): conventional pedometer to record the number of steps and minutes of physical activity performed, and to record the number of resistance exercise sessions performed weekly.	Nutrition information was provided by the application, and participants received weekly feedback consultations about the intervention by telephone.	(a) Android smartphone (b) Smart After-Care app (c) InbodyBand digital pedometer	(i) Drop out: 18%. (ii) Intervention smartphone group: ↑ physical function. (iii) Pedometer control group: ↑ physical function.
Varas et al., 2018 [83]	RCT (8 weeks)	40 (patients with COPD)	68 ± 8	Experimental group (*n* = 17): walking 5 days a week for 30–60 min. Control group (*n* = 16): general recommendations to walk more every day.	Not considered.	(a) OMRON Walking Style X (b) Pocket HJ-320e digital (c) Pedometer	(i) Drop out: 18%. (ii) Experimental group: ↑ endurance shuttle test; ↑ steps/day; ↑ Baecke scores; ↓ total St. George’s Respiratory; ↔ dyspnea; ↔ exacerbation. (iii) Control group: ↔ in all outcomes.
Chokshi et al., 2018 [84]	RCT (24 weeks)	105 (ischemic heart disease patients)	60 ± 11	Incentive arm (*n* = 50): received personalized step goals and daily feedback with remote monitoring for all 24 weeks. Control arm (*n* = 55): usual care with step monitoring.	Not considered.	Misfit Shine	(i) Drop out: 2%. (ii) Incentive arm: ↑ daily steps. (iii) Control arm: ↔ daily steps.
McDermott et al., 2018 [85]	RCT (9 months)	200 (patients with peripheral artery disease)	70 ± 10	Intervention group (*n* = 99): home-based exercise with advice to walk 5 days per week (indoors or outdoors), 10–15 min, up to 50 min per session. Control group (*n* = 101): usual care.	Not considered.	Fitbit Zip	(i) Drop out: 11%. (ii) Intervention group: ↔ 6 min walk distance; ↔ mean steps per day; ↔ mean score for walking impairment questionnaire distance. (iii) Control group: ↔ in all outcomes.
Grossman et al., 2018 [86]	RCT (16 weeks)	11 (obese postmenopausal women)	59 ± 5	Daily energy intake goal between 1200/1500 calories (*n* = 11). High intensity interval training group (*n* = 6): five different 10 min workouts (total body, cardio, lower body, abs, and yoga flex) per 4–5 workouts per week. Endurance group (*n* = 5): walking, jogging, cycling, swimming, or other cardiovascular exercise 60 to 250 min per week.	Both groups followed a calorie-restricted diet.	Fitbit Charge HR	(i) Drop out: 9%. (ii) High intensity interval training group: ↓ fat mass, ↓ BMI; ↓fat free mass. (iii) Endurance group: ↓ Body mass; ↓ BMI; ↓ waist circumference; ↓ average calories consumed.
Tran et al., 2017 [87]	RCT (6 months)	422 (adults with metabolic syndrome)	57 ± 5	Intervention group (*n* = 214): four 2 h education sessions followed by walking aerobic training protocol. Control group (*n* = 203): one session of standard advice.	The intervention group received nutrition program during the four 2 h education sessions.	Yamax SW-200	(i) Drop out: 10%. (ii) Intervention group: ↑ moderate activity participation, walking time and total physical activity; ↑ steps on average on 7 consecutive days; ↓ sitting time. (iii) Control group: ↔ in all outcomes.
Heron et al., 2017 [88]	RCT (6 weeks)	15 (stroke patients)	69 ± 7	Manual group (*n* = 5): standard care and intervention program based on moderate intensity activity. Manual + pedometer (*n* = 5): also received activity tracker, and were encouraged to keep a daily step-count diary. Control group (*n* = 5): received standard post-transient ischemic attack or minor stroke care.	Assessment of adherence to Mediterranean Diet.	Fitbit Charge or pedometer	(i) Drop out: 0%. (ii) Manual group: ↑ physical activity, ↑ 2 min walk distance, ↑ hospital anxiety and depression scores, ↓ hours sitting per day. (iii) Manual + pedometer: ↑ daily steps, ↑ 2 min walk distance, ↑ hospital anxiety and depression scores. (iv) Control group: ↑ 2 min walk distance.
Swartz et al., 2017 [89]	RCT (12 weeks)	40 (obese patients)	62 ± 6	Intervention group (*n* = 20): provided an activity tracker and set a step goal of 7000 steps per day by the end of the intervention. Control group (*n* = 20): usual care.	Not considered.	Jawbone™ Up24	(i) Drop out: 13%. (ii) Intervention group: ↑ steps per week. (iii) Control group: ↔ steps per week.
Jakicic et al., 2016 [90]	RCT (24 months)	470 (obese adults)	18–35 *	Received a behavioral weight loss intervention (*n* = 470). Standard behavioral weight loss intervention (*n* = 233). Technology-enhanced intervention (*n* = 237).	Not considered.	BodyMedia Fit	(i) Drop out: 25%. (ii) Standard behavioral weight loss intervention: ↓ Body mass; ↓ sedentary time, sedentary time, and light-intensity physical activity across time, ↔ fat mass, ↔ lean mass, ↔body fat, ↔bone mineral content, ↔bone mineral density; ↔cardiorespiratory fitness. ↔ total moderate-to-vigorous physical activity. (iii) Technology-enhanced intervention group: ↓ Body mass.

Age values are presented as mean ± standard deviation, unless otherwise noted. * Range reported, rather than standard deviation. Abbreviation: RCT = randomized controlled trial; HR = heart rate; BMI = body mass index; COPD = chronic obstructive pulmonary disease; ↔ = no changes; ↓ = decrease; ↑ = increase.

## Data Availability

Non applicable.
